# Influence of Dietary Astaxanthin on the Hepatic Oxidative Stress Response Caused by Episodic Hyperoxia in Rainbow Trout

**DOI:** 10.3390/antiox8120626

**Published:** 2019-12-06

**Authors:** Carmen Tatiana Kalinowski, Laurence Larroquet, Vincent Véron, Lidia Robaina, María Soledad Izquierdo, Stéphane Panserat, Sachi Kaushik, Stéphanie Fontagné-Dicharry

**Affiliations:** 1 Grupo de Investigación en Acuicultura (GIA), Research Institute in Sustainable Aquaculture and Marine Conservation (IU-ECOAQUA), Universidad de Las Palmas de Gran Canaria, Crta. Taliarte s/n, 35214 Telde, Spain; carmentatiana.kalinowski@fpct.ulpgc.es (C.T.K.); lidia.robaina@ulpgc.es (L.R.); marisol.izquierdo@ulpgc.es (M.S.I.); sachi.kaushik@ulpgc.es (S.K.); 2 NUMEA, INRA, University Pau & Pays Adour, E2S UPPA, 64310 Saint-Pée-sur-Nivelle, France; laurence.larroquet@inra.fr (L.L.); vincent.veron@inra.fr (V.V.); stephane.panserat@inra.fr (S.P.)

**Keywords:** astaxanthin, episodic hyperoxia, liver, oxidative status, rainbow trout

## Abstract

A 13-week feeding trial was carried out with juvenile rainbow trout to test two diets: a control diet without astaxanthin (AX) supplementation (CTRL diet), and a diet supplemented with 100 mg/kg of synthetic AX (ASTA diet). During the last week of the feeding trial, fish were exposed to episodic hyperoxia challenge for 8 consecutive hours per day. Episodic hyperoxia induced physiological stress responses characterized by a significant increase in plasma cortisol and hepatic glycogen and a decrease in plasma glucose levels. The decrease of plasma glucose and the increase of hepatic glycogen content due to episodic hyperoxia were emphasized with the ASTA diet. Hyperoxia led to an increase in thiobarbituric acid-reactive substances in the muscle, diminished by dietary AX supplementation in both liver and muscle. Muscle and liver AX were increased and decreased respectively after 7-day episodic hyperoxia, leading to an increase in flesh redness. This augment of muscle AX could not be attributed to AX mobilization, since plasma AX was not affected by hyperoxia. Moreover, hyperoxia decreased most of antioxidant enzyme activities in liver, whereas dietary AX supplementation specifically increased glutathione reductase activity. A higher mRNA level of hepatic glutathione reductase, thioredoxin reductase, and glutamate-cysteine ligase in trout fed the ASTA diet suggests the role of AX in glutathione and thioredoxin recycling and in de novo glutathione synthesis. Indeed, dietary AX supplementation improved the ratio between reduced and oxidized glutathione (GSH/GSSG) in liver. In addition, the ASTA diet up-regulated glucokinase and glucose-6-phosphate dehydrogenase mRNA level in the liver, signaling that dietary AX supplementation may also stimulate the oxidative phase of the pentose phosphate pathway that produces NADPH, which provides reducing power that counteracts oxidative stress. The present results provide a broader understanding of the mechanisms by which dietary AX is involved in the reduction of oxidative status.

## 1. Introduction

Farmed fish are continuously exposed to stressful conditions induced by physical, chemical, and biological factors such as crowding, handling, changes in diets, and water quality, leading to susceptibility to viral or bacterial infections. Challenging situations generate physiological alterations very much linked to oxidative stress, which is the result of an imbalance between the production of reactive oxygen (ROS) and nitrogen species (RNS) by cell respiration and immune responses, and the state of the antioxidant defenses [1]. In order to protect against oxidative stress, organisms have developed antioxidant systems consisting of low-molecular-mass compounds including glutathione, ascorbic and uric acid, tocopherols, and carotenoids, and high-molecular-mass proteins including superoxide dismutases, catalases, Se-dependent glutathione peroxidases, glutathione reductase, and glucose-6-phosphate dehydrogenase [2].

Carotenoids are natural pigments with immune-stimulant and antioxidant properties [3,4] and are present in the integument of many vertebrate species, generating bright-colored traits [5] Among carotenoids, astaxanthin (AX) is the most commonly used feed additive in order to achieve the characteristic red-pink coloration in crustaceans and salmonids [6,7]. The red coloration of AX is due to the extended chain of conjugated double-bonds at the center of its chemical structure. This chain, including 13 double bonds, is also responsible for the potent antioxidant effect of AX, involved in neutralizing singlet oxygen, scavenging superoxide anions, and hydroxyl radicals [8]. Moreover, it can effectively scavenge lipid radicals and destroy peroxide chain reactions to protect polyunsaturated fatty acids (PUFAs) and sensitive membranes [9].

Besides its role in pigmentation, a few studies have focused on the role of AX on fish health [10], and to our knowledge not much work has been undertaken to assess the potential antioxidant function of this xanthophyll. In rainbow trout, dietary AX enhanced plasma [11], liver, and kidney antioxidant defense [12]. Similarly, in European seabass fry, dietary AX together with sodium taurocholate reduced lipid peroxidation and increased total antioxidant status [13]. In juvenile olive flounder, plasma superoxide dismutase (SOD) activity was lowered by dietary AX [14]. In the characin *Hyphessobrycon callistus*, body SOD and glutathione peroxidase (GPX) activity were decreased by dietary carotenoids (mix of AX and β-carotene) under normal feeding conditions and after an ammonia stress [15,16]. In the latter studies, significant negative correlations were found between body carotenoids (AX, β-carotene) and antioxidant parameters such as SOD and GPX activity [15,16]. In common carp, on the other hand, there was a positive correlation between dietary AX and tissue total antioxidant status and SOD activity before and after ammonia stress [17]. Similarly to common carp, in wild brown trout, intense carotenoid-based skin coloration was found to be closely linked to a high non-enzymatic antioxidant capacity and high activity of hepatic SOD and catalase (CAT) [18].

Based on the hypothesis that dietary AX provides physiological benefits in terms of antioxidant defense, the present study aims to evaluate the homeostatic and stress responses in the liver of this species fed either a control diet (CTRL) or an astaxanthin-supplemented diet (ASTA), and exposed to two environmental conditions, normoxia and episodic hyperoxia, as a stressor causing oxidative stress. Daily oxygen fluctuations in aquaculture systems are quite common and have a more pronounced effect than continuous hyperoxia [19], with compensation being achieved within 24 h [20]. Hence, an episodic hyperoxia regimen was tested in the present study for a period of one week. In addition, as carotenoid-based traits may indicate an individual’s capacity to tackle oxidative stress [21], we also assessed skin and flesh pigmentation as biomarkers of oxidative status in rainbow trout.

## 2. Material and Methods

### 2.1. Diets and Fish

Two iso-nitrogenous, iso-lipidic, and iso-caloric feeds were formulated, manufactured, and tested at the INRA experimental fish farm in Donzacq (Landes, France) (Table 1). The experimental diets differed in AX content as CTRL diet had no AX and the ASTA diet was supplemented with 100 mg of chemically synthesized AX per kilogram of feed, a dietary level considered safe for salmonids according to the European Food Safety Authority [22]. The synthetic AX used was Carophyll Pink^®^ containing 10% AX (DSM Nutrition, Village-Neuf, France). All-female diploid rainbow trout (*Oncorhynchus mykiss*) with an initial weight of 309 ± 10 g were used. Nine 800-L cylindrical fiberglass tanks were stocked with 30 fish each and supplied with flow-through spring water, at 17 °C. Each diet was hand-fed twice a day to visual satiation. Prior to the feeding trial, fish were fed a commercial diet (T3P Omega Skretting, Fontaine-les-Vervins, France). All experimental procedures complied with the European Directive 010/63/EU for the protection of animals used for scientific purposes, and the French Decree no. 2013-118 for animal experimentation.

**Figure 1:**
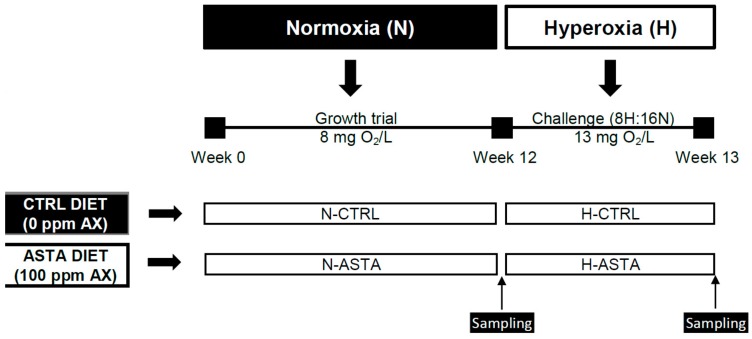
Experimental design. Rainbow trout were fed on a CTRL or ASTA diet during 12 weeks and reared under normoxic conditions. For the last week of the trial, fish were fed the same experimental diets and exposed to an episodic hyperoxia challenge for 8 h and 16 h under normoxia conditions, termed 8H:16N. AX, astaxanthin.

The experimental design is shown in Figure 1. This experiment was conducted for a total period of 13 weeks. During the first 12 weeks of the growth trial, fish were fed on the CTRL or ASTA diet and reared under normoxic conditions (8 mg/L). For the last week of the trial, fish continued with the experimental diets and were exposed to an episodic hyperoxia challenge, termed 8H:16N. For episodic hyperoxic conditions oxygen was increased from 8 to 13 mg/L (163%) and was established from 9:00 to 17:00 for 8 h (8H); afterwards, oxygen levels returned to normoxic conditions for 16 h (16N). A factorial design (2 × 2) was performed to compare the effect of two distinct diets (CTRL and ASTA) and two environmental conditions (normoxia and hyperoxia) on the studied variables. For growth performance parameters such as weight gain, daily growth index, specific growth rate, and feed efficiency, only dietary effect was assessed in normoxic conditions (N-CTRL versus N-ASTA), as the comparison between two different periods of 12 weeks for the growth trial and only 1 week for the episodic hyperoxia challenge was not considered appropriate. Similarly, the impact of episodic hyperoxia on color variables and tissue AX content was assessed only for the ASTA group (N-ASTA versus H-ASTA), as the comparison for non-colored fish was not considered appropriate.

At the end of the feeding trial and episodic hyperoxia challenge, ten fish per replicate tank were individually weighed, measured, and sampled. Parameters of growth performance were assessed after the feeding trial, only to check the status of fish prior to the start of the hyperoxia treatment. Before sampling, fish were feed deprived for 1 day, anaesthetized with benzocaine, and killed with a blow to the head. For whole-body composition analysis, three fish per tank were randomly collected. The rest of the fish were taken for plasma analyses (cortisol, glucose, triglycerides, and AX), skin and muscle color measurements, and liver and viscera weights. Blood was collected from the caudal vein with heparinized syringes. From the seven livers per tank, four were used for determination of AX and thiobarbituric acid-reactive substance (TBARS) contents, whereas the remaining three were for gene expression, glutathione, antioxidant enzyme, and glycogen analyses. Muscle samples were only taken from four fish for AX and TBARS content. All tissues were immediately frozen in liquid nitrogen and then stored at −80 °C until analyses.

### 2.2. Proximate Composition

Proximate compositions of diets and whole fish were determined according to the following procedures: Dry matter (DM) after drying at 105 °C for 24 h, protein (Nx6.25) by the Kjeldahl method after acid digestion, ash by incineration at 550 °C for 16 h, and gross energy in an adiabatic bomb calorimeter. Total lipid was extracted and measured gravimetrically [23] using dichloromethane instead of chloroform. Starch content was determined as glucose by a kit, using the amyloglucosidase/hexokinase/glucose-6-phosphate dehydrogenase method (Invivo Labs, France).

### 2.3. Plasma Cortisol, Glucose, and Triglycerides, and Hepatic Glycogen Determination

Blood samples were collected via caudal vein puncture on anesthetized fish and centrifuged at 3000× *g* for 15 min to isolate plasma that was stored at −80 °C. For plasma cortisol levels, the immunoassay Access Immunoassays System, Cortisol (ref 33600, ©2010 Beckman Coulter, Inc., Indianapolis, IN, USA) was used. The rabbit anti-cortisol antibody and cortisol–HRP conjugate (Fitzgerald Industries International, Concord, MA, USA) were used at a final dilution of 1:25,000 and 1:4000 in coating buffer and EIA Buffer, respectively. Plasma glucose levels were analyzed using an Accu-Chek Advantage glucose meter (Roche, Basel, Switzerland). Plasma triglycerides were determined using the Beckman Coulter AU System Triglyceride procedure based on a series of coupled enzymatic reactions. Hepatic glycogen was determined by a hydrolysis technique [24]. Briefly, each sample was ground in 1 M HCl (VWR, Fontenay-sous-Bois, France). An aliquot was neutralized by 5 M KOH (VWR) and centrifuged 10 min at 10,000× *g* at 4 °C to measure free glucose content in samples using Plasma glucose kit (Glucose RTU, BioMérieux, Marcy-l’Etoile, France) according to the manufacturer’s instructions. Remaining ground tissue was boiled at 100 °C for 2.5 h and then analyzed for total glucose (free glucose + glucose obtained from glycogen hydrolysis) using the same protocol as for the aliquot. Glycogen content was evaluated by subtracting free glucose levels.

### 2.4. Skin and Muscle Color Analysis

Skin color was measured on the left side of the fish and three zones were fixed along the lateral line. For muscle color, a left side fillet was taken and also three zones were established along the central part. From skin and muscle, triplicate measurements were taken at each zone using a tri-stimulus colorimeter CR 400 Minolta. The color measurements taken were in accordance with the recommendations of the International Commission on Illumination [25]: the L*-value represents lightness (L* = 0 for black, L* = 100 for white), the a*-value represents the intensity in red and the b*-value represents the intensity in yellow. A mean from the three zones recorded in skin and muscle were used for color analysis. For correlation analysis the a*-value was chosen since this variable is the most associated with astaxanthin content.

### 2.5. Astaxanthin Extraction and Quantification

The procedure for AX extraction used was as per [26] Briefly, 100 μL of plasma and approximately 50 mg of minced liver and muscle were weighed into Eppendorf tubes. Afterwards, 200 μL of distilled water and 150 μL of ethanol were added. Mixtures were flushed with nitrogen, sonicated for 1 min and vortexed for 5 min. The mixture was then extracted twice with 1 mL of hexane using vortex mixing for 15 min each time. Hexane phases were recovered after centrifuging for 5 min at 2500× *g* (4 °C), combined and evaporated to dryness with a nitrogen flow. The mixture was immediately re-dissolved in adequate volume of chromatographic phase and filtered through a 0.45 μm filter into amber glass vials under nitrogen prior to HPLC injection. AX quantification in liver, muscle, and plasma was carried out according to the method of [27]. An Agilent 1260 Infinity II system equipped with a diode array detector (DAD) and a 150 × 4.60 mm reverse phase C18 Thermo column were used. The mobile phase was 80% MeOH/H_2_O (9:1) and 20% ethyl acetate, at a flow rate of 1.0 mL min^−1^; the injection volume was 10 μL, and the effluent from the column was monitored at a wavelength of 472 nm. Astaxanthin was quantified by an external standard method using a standard curve generated with authentic crystalline astaxanthin (Sigma-Aldrich, Madrid, Spain).

### 2.6. Thiobarbituric Acid Reactive Substances

TBARS were determined according to the protocol of [28] with some modifications. Briefly, 50 μL of 1% (*w*/*v*) butylated hydroxytoluene in ethanol were added to 500 mg of tissue followed by 1.5 mL of 20% (*w*/*v*) trichloroacetic acid and 2.95 mL of 50 mM thiobarbituric acid solution, both freshly prepared. The reagents were mixed in a stoppered test tube and heated at 100 °C for 25 min. After cooling, particulate matter was removed by centrifugation at 2000× *g* (4 °C). Absorbance in the supernatant was determined in a spectrophotometer at 532nm against a blank sample. The concentration of thiobarbituric acid reactive substances (TBARS), expressed as mmol malondialdehyde/g tissue, was calculated using the absorption coefficient of 0.156 μM^−1^ × cm^−1^.

### 2.7. Liver Antioxidant Enzyme Activity

Antioxidant enzyme activities were assayed in liver as described previously [29]. Briefly, superoxide dismutase (SOD, EC 1.15.1.1) activity was measured at 37 °C by monitoring the inhibition of nitrotetrazolium reduction at 550 nm. Catalase (CAT, EC 1.11.1.6) activity was measured at 30 °C by monitoring the decomposition of H_2_O_2_ at 240 nm. Glutathione peroxidase (GPX, EC 1.11.1.9) activity was assayed at 30 °C by the coupled reaction with glutathione reductase (GR) using cumene hydroperoxide and H_2_O_2_ as substrates for measuring total GPX, selenium-dependent GPX (Se-GPX) and non-selenium-dependent GPX (NS-GPX) respectively. GR (EC 1.6.4.2) activity was determined at 30 °C by monitoring NADPH oxidation at 340 nm. Glutathione-S-transferase (GST, EC 2.5.1.18) activity was assayed at 30 °C by following the conjugation of glutathione with 1-chloro-2,4-dinitrobenzene at 340 nm.

### 2.8. Liver Glutathione

Total glutathione (tGSH) and oxidized glutathione (GSSG) were measured in liver using Cayman glutathione assay kit (Bertin Pharma, Montigny-le-Bretonneux, France) according to the manufacturer’s instructions with protein concentration assessed by the method of [30] using bovine serum albumin as a standard. Reduced glutathione (GSH) was calculated as tGSH-GSSG and oxidative stress index (OSI) as 100 × (2GSSG/tGSH).

### 2.9. Gene Expression Assays

Total RNA was isolated from liver by using Trizol reagent (Invitrogen, Cergy-Pontoise, France). Quantitative RT-PCR was performed as described previously [31]. Briefly, complementary DNA was generated from 1 mg total RNA using SuperScriptIII RT (Invitrogen) and a mix of oligo (dT)15 and random primers (Promega, Charbonnières, France). Quantitative PCR analyses were performed with 2 μL of the diluted RT reaction mixture (dilution 40) and 4 μL of master mix added with 0.4 mM of each primer (Table 2). Relative quantification of target gene transcripts were performed using elongation factor 1α (ef1α) as the reference gene and N-CTRL as the reference group using the ΔΔCt method [32].

### 2.10. Statistical Analysis

All the data were presented as means with their standard errors. The number of fish measured for each parameter is specified in tables and figures. Data were tested for normality and homogeneity of the variances with Levene’s test. Two-way ANOVA was used to determine the main effect of the diet factor (D), the environment factor (E), and their interaction (DxE). One-way ANOVA was used for growth performance parameters, color evaluation and tissue AX content, followed by a Student-Newman-Keuls test. Significant differences were accepted at *p* ≤ 0.05. The Pearson correlation coefficient was calculated to analyze the significance of linear relationships between skin and muscle redness (a*) and the studied variables. Correlations with *p* ≤ 0.05 were considered significant. All statistical analyses were performed using SPSS (IBM, Chicago, IL, USA).

## 3. Results

### 3.1. Growth and Whole Body Composition

At the end of the 12-week feeding trial there was no significant effect of the CTRL and ASTA diet on growth performance parameters or whole body composition (Table 3). No mortality was recorded throughout the feeding trial and the 7-day episodic hyperoxia challenge.

### 3.2. Skin and Muscle Color and Liver and Muscle Astaxanthin (AX)

Dietary AX supplementation led to significantly increased skin redness under both normoxia and hyperoxia conditions (Table 4). No significant effect of episodic hyperoxia (N-ASTA versus H-ASTA) was observed in either of the skin color variables. Muscle redness and yellowness were significantly higher in trout fed the ASTA diet under both normoxic and hyperoxic conditions (Table 4). Moreover, episodic hyperoxia (N-ASTA versus H-ASTA) also exerted an increasing effect on these muscle color variables. Muscle lightness was influenced by the diet, with trout fed the ASTA diet having a low value under both normoxic and hyperoxic conditions (Table 4).

Rainbow trout fed the ASTA diet had significantly increased muscle, liver and plasma AX content, under both normoxia and hyperoxia conditions (Table 4). Episodic hyperoxia (N-ASTA versus H-ASTA) had a significant effect only on AX content in the liver with fish exposed to episodic hyperoxia challenge having lower values than in rainbow trout reared under normoxic conditions. Muscle AX content was slightly higher (albeit not significant, *p* = 0.09) in trout exposed to hyperoxic conditions.

### 3.3. Plasma Cortisol, Plasma Glucose, Hepatic Glycogen, and Plasma Triglycerides

**Figure 2:**
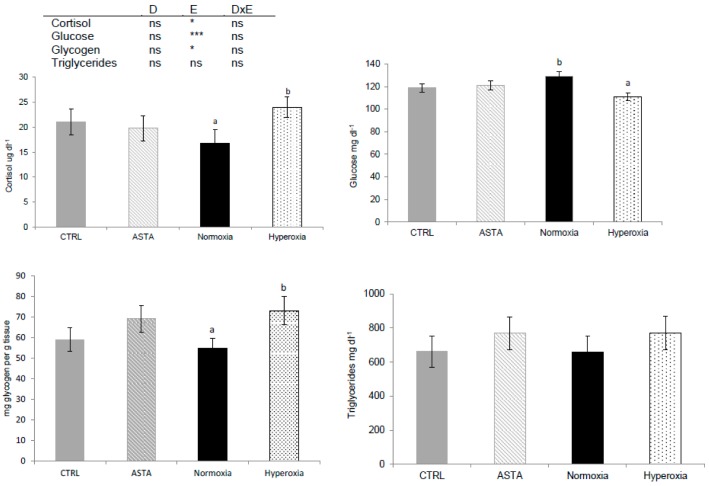
Plasma cortisol (*n* = 9), glucose (*n* = 21), hepatic glycogen (*n* = 9), and triglycerides (*n* = 12) of CTRL and ASTA fed rainbow trout exposed to normoxic (12 weeks) and episodic hyperoxic (1 week) environments. Values are presented as means ± SEM. Different superscript letters denote significant differences between factors determined by two-way ANOVA (*p* < 0.05). D, diet factor; E, environment factor; DxE, interaction between diet and environment; * *p* < 0.05; *** *p* < 0.001; ns not significant.

Plasma cortisol, plasma glucose and hepatic glycogen were only significantly affected by environmental conditions (Figure 2). Episodic hyperoxia increased plasma cortisol but decreased plasma glucose. The lowering of plasma glucose, appeared to be enhanced, but not significant (*p* = 0.09), by the interaction between diet and environment, as the lowest plasma glucose values were observed in fish fed the ASTA diet and exposed to hyperoxia. Liver glycogen was higher with hyperoxic conditions than with normoxia treatment. The highest liver glycogen content was found in H-ASTA fish, similar to the lowest plasma glucose content. Plasma triglycerides were not significantly affected by any of the factors evaluated (Figure 2).

### 3.4. Thiobarbituric Acid-Reactive Substance (TBARS) in Flesh and Liver

**Figure 3:**
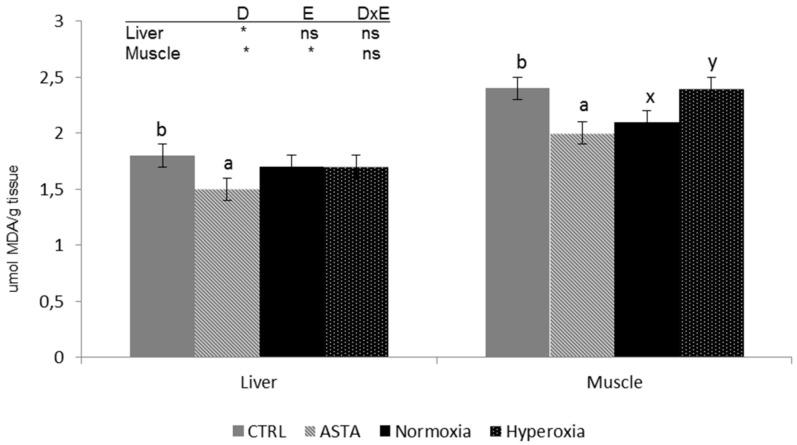
Liver and muscle thiobarbituric acid-reactive sustances (TBARS) (*n* = 12) of CTRL and ASTA-fed rainbow trout exposed to a normoxic (12 weeks) and episodic hyperoxic (1 week) environment. Values are presented as means ± SEM. Different superscript letters (^a,b^ for diet factor and ^x,y^ for environment factor) within a tissue denote significant differences between factors determined by two-way ANOVA (*p* < 0.05). D, diet factor; E, environment factor; DxE, interaction between diet and environment; * *p* < 0.05; ns not significant.

Analysis of lipid peroxidation products in muscle and liver showed differences between the two tissues, with the highest TBARS content recorded in muscle (Figure 3). TBARS values in both tissues were significantly affected by the dietary factor with lower values in rainbow trout fed the ASTA diet than in fish fed the CRTL diet. Moreover, in the muscle, the environment also affected TBARS content, with normoxia-exposed fish displaying higher values than hyperoxia-exposed individuals.

### 3.5. Hepatic Antioxidant Enzyme Activity

Environmental conditions tested had a more profound effect on the activities of hepatic antioxidant enzymes than dietary conditions (Table 5). The antioxidant enzymes CAT, NS-GPX, SOD, GR, and GST were significantly reduced by the hyperoxia challenge. Only the activity of hepatic GR was influenced by the diet, as trout fed the ASTA diet had an increased activity of this enzyme. The other antioxidant enzymes, total-GPX and Se-GPX, were not influenced by any of the factors tested.

### 3.6. Hepatic Glutathione

Contrary to results found on hepatic antioxidant enzymes, two-way ANOVA revealed that the dietary factor had a more significant effect on hepatic glutathione than the environmental factor (Table 6). Rainbow trout fed the ASTA diet had significantly lower liver GSSG, resulting in an improved ratio between reduced and oxidized glutathione (GSH/GSSG), and better OSI.

### 3.7. Hepatic Gene Expression

Of the hepatic oxidative stress-related genes studied (Table 7), mRNA levels of glutathione reductase (*gr*), glutamate-cysteine ligase catalytic subunit (*gclc*), and thioredoxin reductase (*tr*) showed a clear effect of diet, with a higher expression of the above-mentioned genes in rainbow trout fed the ASTA diet than fish fed a carotenoid-depleted diet. However, the environmental factor exerted the opposite effect to the dietary factor on other genes such as glutathione peroxidase 1a (*gpx1a*), selenoprotein 1 (*sepp1*), and nuclear factor kappa-light chain enhancer of activated beta cells inhibitor (*iκb*), with reduced expression in hypoxia-exposed fish compared to normoxia-exposed fish. The rest of oxidative stress related genes studied did not elicit a specific response to diet or environment. With glucose metabolism-related genes (Table 8), two-way ANOVA highlighted that both environment and diet exerted a significant effect on glucose-6-phosphate dehydrogenase (*g6pd*), hyperoxia having a down-regulating effect, and the ASTA diet an up-regulating effect. Glucokinase b (*gckb*) was only higher expressed in the ASTA diet compared to CTRL diet. Concerning phosphoglucomutase1X2 (*pgm1X2*) and 1X3 (*pgm1X3*), episodic hyperoxia lowered the mRNA levels in comparison to normoxia conditions. The other glucose metabolism related genes were not significantly affected by the factors studied.

### 3.8. Skin and Muscle Redness Correlation

In this study, we detected a positive, strong and significant correlation between muscle redness and hepatic AX content; however, with skin redness although the correlation was also significant, it was found to be moderate (Table 9). A positive, significant, but weak correlation was found between muscle redness and hepatic OSI/hepatic *gr*, *gclc*, *tr*, *g6pd*, *gckb* mRNA levels, with no correlation with hepatic GR activity found (Table 9). Skin redness presented no correlation with hepatic OSI and hepatic *gclc* mRNA level, but showed a positive and significant although weak correlation with hepatic GR activity/hepatic *gr*, *tr*, and *g6pd* mRNA levels. Negative, weak, and significant correlations were only observed among muscle and skin redness and hepatic and muscle TBARS. As reported in Table 9, muscle redness presented higher and more significant associations among the dependent variables evaluated than skin redness.

## 4. Discussion

Carotenoids are part of a complex and integrated antioxidant defense system and their biological effects are mostly a result of co-operative interactions with endogenous and exogenous antioxidants, rather than a direct antioxidant effect [33]. In the present study, dietary AX supplementation did not affect the growth performance of juvenile rainbow trout. Similarly, no effect of dietary AX on growth was reported in rainbow trout [34] or in other fish species fed 20–100 mg AX/kg [17,35,36].

### 4.1. Dietary AX and Episodic Hyperoxia on Physiological Response

Plasma cortisol levels found in this study indicate that hyperoxia caused a stress response in rainbow trout. Cortisol values in resting or unstressed fish generally range between 0.5–4.0 µg dL^−1^, while in stressed fish, the values increase up to 10–20 µg dL^−1^ [37,38,39]. Our data on elevated cortisol values in rainbow trout exposed to episodic hyperoxia are also in agreement with the data of [19]. However, it is important to mention that the values found under normoxic conditions in this study are also high. This could be attributed to the time employed for the fish capture before anesthesia. The triggering of metabolic adjustments due to stressors also raises energy demands [40], increasing glycogen mobilization as reflected by increased plasma glucose levels [41]. Hepatic glycogen is also considered as a metabolic indicator of secondary stress response [42], which is decreased in order to mobilize glucose to peripheral tissues. In contrast, in the present study the 7-day hyperoxia challenge did not induce the glucose stress response normally observed in fish, but instead decreased plasma glucose and increased hepatic glycogen content. Moreover, the hypoglycemic effect of episodic hyperoxia noticed in this trial was associated with decreased mRNA expression of phosphoglucomutase, an enzyme involved in both glycogen synthesis and mobilization. However, to date there are very few studies in fish dealing with oxygen availability and the dynamics of cellular energy metabolism when the partial pressure of oxygen increases. The decrease of plasma glucose and the increase of hepatic glycogen content due to episodic hyperoxia were emphasized with the ASTA diet. In Asian seabass, dietary AX showed a hypoglycemic effect, suggesting that this carotenoid may be possibly beneficial in stimulating the insulin sensitivity of fish [43]. Similarly, a meta-analysis to evaluate the efficacy of AX supplementation on plasma lipid and glucose concentrations revealed a slight glucose-lowering effect of this carotenoid in humans [44]. Concerning hepatic glycogen, a glycogen-sparing effect of AX was found in mice under situations of prolonged exercise [45]. There is evidence that dietary AX promotes lipid metabolism in mice [46]. Thus, the effect of dietary AX on lipid metabolism in rainbow trout deserves further investigation. In fact, it has been found that dietary carotenoids have an impact on lipid profile of rainbow trout liver, potentially due to their antioxidant functions [47].

Given that lipid peroxidation is reported to be a major contributor to the loss of cell function [48], fighting against lipid peroxides is a key issue in aquatic species due to their generally higher tissue concentrations of n-3 long-chain PUFAs than other animal species. Lipid peroxidation products such as TBARS are considered pertinent markers of lipid peroxidative stress [49]. In this study, the lipid peroxidation level was 30% higher in muscle than in the liver, and the episodic hyperoxia challenge affected lipid peroxidation only in the muscle and not in the liver. A study on goldfish showed a short-lived increase in TBARS levels during early hours of hyperoxia in most tissues evaluated, but they quickly returned to values near or significantly below control levels [50]. However chronic hyperoxia condition tested in Atlantic salmon pre-smolt for 6 weeks significantly increased liver TBARS [51]. Contrary to episodic hyperoxia challenge, dietary AX supplementation decreased susceptibility to lipid peroxidation in both liver and muscle. Similarly, dietary AX decreased TBARS concentration in muscle of rainbow trout [52] and liver of yellow catfish submitted to crowding stress [53].

It has been suggested that increased oxidative stress leads to AX mobilization in Atlantic salmon [54]. We did not observe any effect of the episodic hyperoxia challenge on skin color or plasma AX levels, although there was an improvement with dietary AX supplementation. These results suggest that there is no allocation conflict between rainbow trout skin coloration and internal antioxidant response after episodic hyperoxia. In contrast to skin color results, muscle redness and AX content were significantly increased by hyperoxia in ASTA-fed rainbow trout, denoting a higher AX allocation to this tissue, which is an important site of AX accumulation during the growth phase [55]. The increased muscle AX deposition together with higher TBARS levels recorded after the hyperoxia challenge may suggest a high susceptibility of the muscle tissue to oxidative damage. Showing an opposite trend to muscle, liver displayed a lower AX content after episodic hyperoxia. This could be related to the higher antioxidant enzyme activity in this tissue, as indicated by the more efficient scavenging of peroxyl radicals reflected by the non-increase of TBARS in liver.

### 4.2. Control of Antioxidant Enzymes by Episodic Hyperoxia and Dietary AX

Among ROS-induced adjustments of antioxidants to hyperoxia, endogenous enzyme activity was significantly reduced, with the exception of total GPX and Se-GPX. The reduced activity found in this study could be due to the increasing amounts of ROS generated by 7-day episodic hyperoxia, which may have overwhelmed the response of endogenous antioxidant enzymes. Our results are in accordance with a study in Atlantic salmon that found a decrease of SOD and Se-GPX activity in response to 6-week moderate hyperoxia [51]. Transient activation of antioxidant enzymes seems to take place in gills and liver of acute hyperoxia-exposed rainbow trout [56]. Hence, the exposure time to hyperoxia may determine the activation of endogenous antioxidant enzymes, as these are considered the first level of antioxidant defense [33]. The activities of antioxidant enzymes could partly result from induction of the gene expressions of antioxidant enzymes, regulated in fish by the nuclear factor erythroid 2-related factor 2 [29]. However, neither antioxidant enzyme mRNA levels, except *gpx1a* and *sepp1*, nor mRNA level of *nrf2* were influenced by the environmental conditions tested. Similarly to our findings, the levels of hepatic *sod1*, *cat,* and *gpx* mRNA of Atlantic salmon exposed to hyperoxia for a prolonged period of time did not change in comparison to the levels under normoxia [57]. However, contrary to our results, Atlantic cod exposed to 145% O_2_ saturation for 6 weeks showed upregulated hepatic *gpx* mRNA levels compared to the normoxia group [58]. Elevation in the expression of hepatic antioxidant-related proteins was observed in the flatfish *Solea senegalensis* in response to 223% O_2_ saturation [59].

Concerning the effect of dietary AX supplementation on endogenous antioxidant enzymes (both in terms of activities and mRNA level of GR), the enzyme involved in the reduction of GSSG to GSH using NADPH as a reducing cofactor [60] was increased, whereas other antioxidant enzymes were not significantly affected. Similar to our results, an enhanced GR activity was reported in liver of rainbow trout after 8 weeks of AX supplementation [12].

### 4.3. Control of Glutathione Metabolism by Episodic Hyperoxia and Dietary AX

No significant effect of episodic hyperoxia was observed on the glutathione response, in contrast to [1] who suggested an increase of cellular GSH during stress conditions, but similarly to data from Atlantic cod [58]. In a study with rainbow trout also exposed to episodic hyperoxia for 12 h and 12 h of normoxia (12HYP:12NOR), total GSH was increased without a simultaneous increase in hepatic GSSG, and hence this could be regarded as a sign of enhanced potential defense against ROS and not as a sign of oxidative stress [19].

AX supplementation tested in this study enhanced the glutathione response, with a decreased liver GSSG, increased GSH/GSSG ratio, and decreased OSI. The significant increase of GSH/GSSG could be related to the fact that glutathione is recycled in a NADPH-dependent reaction by GR, and the GR activity was significantly increased in fish fed the ASTA diet. Similarly, in a study with rainbow trout fed an AX-supplemented diet for 8 weeks, elevated liver GR activity allowed liver GSH amounts to be sustained [12]. In addition, NADPH synthesis seems to be augmented by the ASTA diet through the increase in g6pd mRNA level. Among enzymes able to produce NADPH, G6PD is considered the most important one [61], catalyzing the rate-limiting step in the oxidative pentose phosphate pathway (oxPPP) that provides NADPH [62]. Dietary AX also boosted the oxPPP through the upregulation of glucokinase (GK), a glucose-phosphorylating enzyme that yields glucose 6-phosphate (G6P), the main substrate for oxPPP. Consequently, the overall improvement of hepatic glutathione redox status in rainbow trout fed the ASTA diet is possibly due to the increase of G6P and the increased activity of G6PD, both key participants in the synthesis of NADPH necessary to maintain a proper GSH/GSSG redox balance [63]. An enhancement of the liver glutathione system is of pivotal importance, since in hepatocytes a substantial portion of the intracellularly recycled or synthesized GSH may be exported out of the cells, as the liver is the main GSH producer and storage organ which supplies other tissues [64]. Besides, AX seems to also enhance GSH production, as the gene expression of liver glutamate cysteine ligase (*gclc*), a rate-limiting enzyme for glutathione synthesis [65], was also upregulated. However, the authors of [66] did not find an AX effect on mRNA levels of *gclc* (glutamate-cysteine ligase, catalytic subunit) in cultured hepatocytes.

In addition to the effect on glutathione metabolism, the ASTA diet also up-regulated thioredoxin reductase (*tr*), part of the thioredoxin system that also constitutes another important system to sustain the intracellular redox state [67]. Similarly to the glutathione system, the recycling occurs in a NADPH-dependent reaction by thioredoxin reductase.

### 4.4. Antioxidant Defenses and Tissue Color

In a variety of animals, expression of color traits predicts aspects of performance to resist oxidative stress [21]. It has been suggested that carotenoid-based colorations may signal the availability of other non-pigmentary antioxidants [18]. The correlational approach used in this study showed that both liver non-enzymatic and enzymatic antioxidant response were significantly associated with both skin and muscle redness a*-values. To date, no study has investigated the relationship between carotenoid-based skin and muscle color and oxidative state in rainbow trout. In a study with wild brown trout, carotenoid-based skin colorations were considered a signal of individual quality in terms of antioxidant defenses [18]. Results of this trial in rainbow trout suggest that although both muscle and skin color were associated to tissue AX content and liver antioxidant response, the correlations were higher and more significant in the muscle.

## 5. Conclusions

Our overall results suggest that supplementation with 100 mg kg^−1^ of synthetic AX can improve antioxidant capacity through the role on the expression of certain antioxidant-related genes. The 7-day episodic hyperoxia challenge appeared to induce mild stress, with reduced utilization of glycogen as energy substrate and certain beneficial effects such as the enhancement of flesh redness in rainbow trout. Nevertheless, more research needs to be carried out to elucidate both findings, specifically as this is the first fish study that suggests the role of AX in glutathione and thioredoxin recycling and in glutathione synthesis as well as in pentose phosphate pathway activation in rainbow trout liver.

## Figures and Tables

**Table 1:** Formulation and composition of the experimental diets.

Diet	CTRL	ASTA
Norwegian herring meal ^a^	23	23
Wheat gluten meal ^b^	10	10
Soybean meal ^c^	20	20
Rapeseed meal ^c^	10	10
Fish oil ^a^	19	19
Whole wheat meal ^c^	11.8	11.8
Dibasic calcium phosphate	2	2
Soybean lecithin ^d^	2	2
Vitamins premix ^e^	1	1
Minerals premix ^f^	1	1
Cellulose	0.2	0.1
Free astaxanthin ^g^	-	0.1
Proximate composition		
Dry matter (DM, %)	97.4	97.3
Crude protein (% DM)	41.8	40.8
Total lipid (% DM)	23.7	22.9
Starch (% DM)	7.4	9.1
Ash (% DM)	9.6	9.2
Gross energy (kJ g^−1^ DM)	24.1	23.9

^a^ Norse LT94, crude fish oil and Estrilvo from Sopropêche (Wimille, France). ^b^ Roquette (Lestrem, France). ^c^ Sud-Ouest Aliment (Haut-Mauco, France). ^d^ Louis François (Croissy-Beaubourg, France). ^e^ Vitamin premix (IU or g/kg premix): retinyl acetate, 500,000 IU; cholecalciferol, 250,000 IU; DL α-tocopheryl acetate, 5000 IU; sodium menadione bisulfate, 1 g; thiamin-HCl, 0.1 g; riboflavin, 0.4 g; niacin, 1 g; d-calcium pantothenate, 2 g; pyridoxine-HCl, 0.3 g; d-biotin, 20 mg; folic acid, 0.1 g; cyanocobalamin, 1 mg; l-ascorbyl-2-polyphosphate, 5 g; myo-inositol, 30 g; choline, 100 g. All ingredients were diluted with α-cellulose. ^f^ Mineral mixture (g/kg premix): CaHPO_4_·2H_2_O, 500; CaCO_3_, 215; MgO, 124; KCl,90; NaCl, 40; FeSO_4_·7H_2_O, 20; ZnSO_4_·7H_2_O, 4; MnSO4·H_2_O, 3; CuSO_4_·5H_2_O, 3; NaF, 1; KI, 0.04; Na_2_SeO_3_, 0.03; CoCl_2_·6H_2_O, 0.02. ^g^ Carophyll Pink 10% DSM. ASTA, diet supplemented with 100 mg/kg of synthetic astaxanthin; CTRL, control diet without astaxanthin supplementation.

**Table 2:** Sequences of the PCR primers used to assay gene expression by real-time quantitative polymerase chain reaction.

Gene	Accession Number	Forward Primer Sequence	Reverse Primer Sequence	Amplicon Size
*sod1*	AF469663.1	tggtcctgtgaagctgattg	ttgtcagctcctgcagtcac	201
*sod2*	CA352127.1	tccctgacctgacctacgac	ggcctcctccattaaacctc	201
*cat*	BX087110.3	tgatgtcacacaggtgcgta	gtgggctcagtgttgttgag	195
*gpx1a*	HE687021.1	aatgtggcgtcactctgagg	caattctcctgatggccaaa	131
*gpx4b*	CA344428.1	ttggaggtcaggagccaggt	accctttcccttgggctgtt	152
*gr*	HF969248.1	ctaagcgcagcgtcatagtg	acacccctgtctgacgacat	108
*gclc*	GSONMT00065033001	caaccaactggcagacaatg	cctttgacaaggggatgaga	189
*gstπ*	BX302932.3	tcgctgactggacgaaagga	cgaaggtcctcaacgccatc	196
*tr*	HF969247.1	acaaaatcaaggcgaccaac	ggcagagagaacaggtcgtc	148
*sepp1*	EE605178	gcccaaacaggaagatgtgt	gggcagggagatatggtagg	100
*nrf2*	CA360709.1	tgagctgcagcaatgtctga	gttgggcaatgggtagaagc	124
*keap1α*	GSONMT00034445001	gctacgtgatgtctgcccct	ggtacctcatagcggccagt	116
*nfκb*	BX880658.3	cagcgtcctaccaggctaaagagat	gctgttcgatccatccgcactat	181
*iκbα*	BT074199.1	agagacagactgcgctccac	cggccttcagtagcctctct	72
*g6pd*	EF551311.1	ctcatggtcctcaggtttg	agagagcatctggagcaagt	177
*gcka*	GSONMT00033781001	ctgcccacctacgtctgt	gtcatggcgtcctcagagat	174
*gckb*	GSONMT00012878001	tctgtgctagagacagccc	cattttgacgctggactcct	150
*pgm1X1*	GSONMT00077832001	gaagagagtttcggcacagg	cctccacactctgcttcctc	106
*pgm1X2*	GSONMT00077952001	aaagcatggcttcttcgtca	tggacaatgtggctaaagcc	148
*pgm1X3*	GSONMT00016899001	tgatggtgacggtgatcgta	ggctttagccacattgtcca	181
*g6pcb1*	GSONMG00066036001 ^a^	agggacagttcgaaaatggag	ccagagagggaagaagatgaag	138
*g6pcb2*	GSONMG00013076001 ^b^	cctgcggaacaccttctttg	tcaatttgtggcgctgatgag	195
*ef1α*	AF498320.1	tcctcttggtcgtttcgctg	acccgagggacatcctgtg	159

sod, superoxide dismutase; cat, catalase; gpx, glutathione peroxidase; gr, glutathione reductase; gclc, glutamate-cysteine ligase catalytic subunit; gstπ, glutathione S-transferase π; tr, thioredoxin reductase; sepp1, selenoprotein 1; nrf2, nuclear factor erythroid-2 related factor 2; keap1, Kelch-like ECH-associated protein 1; nfκb, nuclear factor kappa-light chain-enhancer of activated B cells; iκbα, nuclear factor kappa-light chain-enhancer of activated B cells inhibitor α; g6pd, glucose 6 phosphate dehydrogenase; gck, glucokinase; pgm1, phosphoglucomutase1; g6pc, glucose 6 phosphatase; EF1α, elongation factor 1α. ^a^ and also GSONMG00076841001. ^b^ and also GSONMG00014864001.

**Table 3:** Growth performance and final whole-body composition of CTRL and ASTA-fed rainbow trout during 12 weeks under normoxic conditions.

Environment	Normoxia	
Diet	CTRL	ASTA
Final weight (g)	848 ± 50	872 ± 50
DGI ^a^	3.2 ± 0.2	3.3 ± 0.2
SGR ^b^	1.2 ± 0.1	1.2 ± 0.1
VSI ^c^	14.9 ± 1.2	11.4 ± 1.2
HSI ^d^	1.3 ± 0.1	1.0 ± 0.1
CF ^e^	1.9 ± 0.0	1.9 ± 0.0
FCR ^f^	1.1 ± 0.1	1.1 ± 0.1
Whole-body composition		
DM (%)	36.0 ± 0.6	36.6 ± 0.3
Crude protein (%)	17.1 ± 0.2	16.6 ± 0.1
Total lipid (%)	16.9 ± 0.7	17.7 ± 0.1
Ash (%)	1.9 ± 0.1	2.0 ± 0.1

Values are presented as means ± SEM. Different superscript letters within a row denote significant differences among treatments determined by two-way ANOVA (*p* < 0.05). * *p* < 0.05; *** *p* < 0.001. ^a^ DGI, daily growth index = 100 × ((final mean body weight)^1/3^ − (initial mean body weight)^1/3^)/duration; ^b^ SGR, specific growth rate = 100 × ((Ln final mean body weight) − (Ln initial mean body weight))/duration ^c^ VSI, viscerosomatic index = (viscera weight, g/weight of fish, g) × 100; ^d^ HSI, hepato-somatic index = (liver weight, g/weight of fish, g) × 100; ^e^ CF, condition factor = (weight of fish, g)/(length of fish, cm)^3^; ^f^ FCR, feed conversation ratio = dry feed intake/wet weight gain.

**Table 4:** Skin (*n* = 21) and muscle (*n* = 21) color, and tissue astaxanthin content (*n* = 12, ug g^−1^) of CTRL and ASTA-fed rainbow trout exposed to normoxic (12 weeks) and hyperoxic (1 week) environments.

Environment	Normoxia		Hyperoxia	
Diet	N-CTRL	N-ASTA	H-CTRL	H-ASTA
Skin				
Lightness (L*)	54.7 ± 2.2	55.5 ± 1.5	57.9 ± 2.0	54.4 ± 2.1
Redness (a*)	6.8 ± 0.3 ^b^	10.3 ± 0.5 ^a^	6.7 ± 0.4 ^b^	10.3 ± 0.4 ^a^
Yellowness (b*)	1.7 ± 0.7	0.5 ± 0.6	1.4 ± 0.5	1.7 ± 0.6
Muscle				
Lightness (L*)	42.5 ± 0.4 ^a^	35.5 ± 0.4 ^b^	42.2 ± 0.5 ^a^	34.7 ± 0.4 ^b^
Redness (a*)	1.5 ± 0.2 ^b^	12.4 ± 0.4 ^a,B^	1.2 ± 0.1 ^b^	15.2 ± 0.3 ^a,A^
Yellowness (b*)	1.6 ± 0.3 ^b^	8.2 ± 0.5 ^a,B^	2.6 ± 0.3 ^b^	13.3 ± 0.4 ^a,A^
Astaxanthin				
Muscle	0.1 ± 0.0 ^b^	4.9 ± 0.4 ^a^	0.1 ± 0.0 ^b^	6.0 ± 0.6 ^a^
Liver	0.1 ± 0.0 ^b^	1.4 ± 0.13 ^a,B^	0.0 ± 0.0 ^b^	1.1 ± 0.1 ^a,A^
Plasma	0.1 ± 0.0 ^b^	5.9 ± 0.4 ^a^	0.1 ± 0.0 ^b^	5.8 ± 0.6 ^a^

Values are presented as means ± SEM. Different superscript lowercase letters (^a,b^) denote significant differences among CTRL and ASTA-fed rainbow trout exposed to normoxic or hyperoxic environments, and different superscript capital letters (^A,B^) denote significant differences among N-ASTA and H-ASTA determined by one-way ANOVA (*p* < 0.05).

**Table 5:** Liver antioxidant enzyme activity (*n* = 9) of CTRL and ASTA fed rainbow trout exposed to a normoxic (12 weeks) and episodic hyperoxia (1 week) environment.

Antioxidant Enzymes	Diet		Environment		Two way ANOVA		
	CTRL	ASTA	Normoxia	Hyperoxia	D	E	DxE
CAT (U mg pt^−1^)	1152.9 ± 85.4	1227.7 ± 85.7	1307.4 ± 95.8 ^a^	1073.2 ± 63.3 ^b^	ns	*	ns
Total GPX (mU mg pt^−1^)	39.7 ± 2.9	42.0 ± 2.5	43.6 ± 2.7	38.3 ± 2.5	ns	ns	ns
Se-GPX (mU mg pt^−1^)	23.0 ± 2.0	23.8 ± 2.0	22.4 ± 2.5	24.4 ± 1.3	ns	ns	ns
NS-GPX (mU mg pt^−1^)	16.7 ± 1.8	18.1 ± 2.6	21.3 ± 2.3 ^a^	13.5 ± 1.7 ^b^	ns	*	ns
SOD (U mg pt^−1^)	51.6 ± 3.6	54.0 ± 3.8	59.7 ± 2.9 ^a^	45.9 ± 3.7 ^b^	ns	**	ns
GR (mU mg pt^−1^)	9.6 ± 0.5 ^a^	11.8 ± 0.7 ^b^	11.9 ± 0.6 ^y^	9.5 ± 0.7 ^x^	**	**	ns
GST (mU mg pt^−1^)	705.2 ± 34.6	715.1 ± 39.1	762.7 ± 38.6 ^a^	657.6±30.3 ^b^	ns	*	ns

Values are presented as means ± SEM. Different superscript letters (^a,b^) denote significant differences among factors determined by two-way ANOVA (*p* < 0.05). D, diet factor; E, environment factor; DxE, interaction between diet and environment; CAT, catalase, GPX, glutathione peroxidase; SOD, superoxide dismutase; GR, glutathione reductase; GST, glutathione-S-transferase; Se-GPX, selenium-dependent GPX; NS-GPX, non-selenium-dependent GPX. * *p* < 0.05; ** *p* < 0.01.

**Table 6:** Liver total glutathione (tGSH; mmolg^−1^; *n* = 9), oxidized glutathione (GSSG; mmolg^−1^; *n* = 9), reduced glutathione (GSH; mmolg^−1^; *n* = 9), and oxidative stress index (OSI) of CTRL and ASTA-fed rainbow trout exposed to a normoxic (12 weeks) and episodic hyperoxic (1 week) environment.

Glutathione	Diet		Environment		Two way ANOVA		
	CTRL	ASTA	Normoxia	Hyperoxia	D	E	DxE
tGSH	1.70 ± 0.04	1.69 ± 0.04	1.68 ± 0.04	1.71 ± 0.04	ns	ns	ns
GSSG	0.35 ± 0.01 ^b^	0.32 ± 0.01 ^a^	0.33 ± 0.01	0.34 ± 0.01	*	ns	ns
GSH	1.35 ± 0.03	1.37 ± 0.04	1.35 ± 0.04	1.37 ± 0.03	ns	ns	ns
GSH/GSSG	3.96 ± 0.16 ^a^	4.37 ± 0.13 ^b^	4.21 ± 0.18	4.12 ± 0.13	*	ns	ns
OSI	40.97 ± 1.2 ^b^	37.68 ± 1.0 ^a^	39.11 ± 1.31	39.54 ± 1.01	*	ns	ns

Values are presented as means ± SEM. Different superscript letters (^a,b^) denote significant differences among treatments determined by two-way ANOVA (*p* < 0.05). D, diet factor; E, environment factor; DxE, interaction between diet and environment; * *p* < 0.05. OSI = 100 × (2GSSG/tGSH).

**Table 7:** Expression of genes involved in antioxidant response of rainbow trout liver fed two experimental diets (CTRL and ASTA) under normoxic (12 weeks) and episodic hyperoxic (1 week) conditions.

Gene	Diet		Environment		Two-way ANOVA		
	N-CTRL	N-ASTA	Normoxia	Hyperoxia	D	E	DxE
**Antioxidant enzymes**							
*sod1*	1.3 ± 0.2	1.2 ± 0.1	1.1 ± 0.1	1.4 ± 0.2	ns	ns	ns
*sod2*	1.0 ± 0.1	1.1 ± 0.1	1.1 ± 0.1	1.0 ± 0.1	ns	ns	ns
*cat*	1.1 ± 0.1	1.1 ± 0.1	1.1 ± 0.1	1.2 ± 0.1	ns	ns	ns
*gpx1a*	1.0 ± 0.1	1.1 ± 0.1	0.9 ± 0.0 ^a^	1.0 ± 0.1 ^b^	ns	*	ns
*gpx4b*	0.9 ± 0.2	1.3 ± 0.1	1.1 ± 0.1	1.0 ± 0.1	ns	ns	ns
*gr*	1.0 ± 0.1 ^a^	1.4 ± 0.1 ^b^	1.2 ± 0.1	1.1 ± 0.2	**	ns	ns
*gclc*	1.0 ± 0.1 ^a^	1.3 ± 0.1 ^b^	1.2 ± 0.1	1.1 ± 0.1	*	ns	ns
*gstπ*	1.0 ± 0.1	1.1 ± 0.1	1.0 ± 0.1	1.1 ± 0.1	ns	ns	ns
*tr*	1.2 ± 0.1 ^a^	1.6 ± 0.1 ^b^	1.3 ± 0.1	1.5 ± 0.1	*	ns	ns
*sepp1*	0.9 ± 0.1	0.9 ± 0.1	1.0 ± 0.1 ^a^	0.8 ± 0.1 ^b^	ns	*	ns
**Transcription factors**							
*nrf2*	1.0 ± 0.1	1.1 ± 0.1	1.0 ± 0.1	1.0 ± 0.1	ns	ns	ns
*keap1α*	1.2 ± 0.1	1.3 ± 0.1	1.2 ± 0.1	1.3 ± 0.2	ns	ns	ns
*nfκb*	1.0 ± 0.1	1.0 ± 0.1	1.1 ± 0.1	1.0 ± 0.1	ns	ns	ns
*iκbi*	1.0 ± 0.1	1.0 ± 0.1	1.2 ± 0.1 ^b^	0.9 ± 0.1 ^a^	ns	*	ns

Values are presented as means ± SE. Different superscript letters (^a,b^) denote significant differences among treatments determined by two-way ANOVA (*p* < 0.05). D, diet factor; E, environment factor; DxE, interaction between diet and environment; * *p* < 0.05; ** *p* < 0.01.

**Table 8:** Expression of genes involved in glucose metabolism of rainbow trout liver fed two experimental diets (CTRL and ASTA) under normoxic (12 weeks) and hyperoxic (1 week) conditions.

Gene	Diet		Environment		Two-way ANOVA		
	CTRL	ASTA	Normoxia	Hyperoxia	D	E	DxE
**Glucose metabolism**							
*g6pd*	1.0 ± 0.1 ^a^	1.5 ± 0.1 ^b^	1.4 ± 0.1 *^y^*	1.1 ± 0.1 *^x^*	**	*	ns
*gcka*	38.0 ± 21.3	72.3 ± 27.6	45.1 ± 26.0	65.2 ± 23.1	ns	ns	ns
*gckb*	2.4 ± 0.7 ^a^	7.3 ± 1.7 ^b^	5.9 ± 1.6	3.6 ± 1.0	**	ns	ns
*pgm1X1*	1.0 ± 0.1	1.3 ± 0.2	1.2 ± 0.2	1.1 ± 0.2	ns	ns	ns
*pgm1X2*	0.9 ± 0.1	0.9 ± 0.1	1.1 ± 0.1	0.7 ± 0.1	ns	**	ns
*pgm1X3*	0.9 ± 0.1	0.9 ± 0.1	1.1 ± 0.1	0.8 ± 0.1	ns	**	ns
*g6pcb1*	1.3 ± 0.2	1.0 ± 0.1	1.0 ± 0.2	1.2 ± 0.2	ns	ns	ns
*g6pcb2*	1.9 ± 0.5	2.9 ± 0.6	2.2 ± 0.7	2.6 ± 0.5	ns	ns	ns

Values are presented as means ± SE. Different superscript letters (^a,b^ for diet factor and ^x,y^ for environment factor) denote significant differences between factors determined by two-way ANOVA (*p* < 0.05). D, diet factor; E, environment factor; DxE, interaction between diet and environment; * *p* < 0.05; ** *p* < 0.01; *** *p* < 0.001.

**Table 9:** Correlation coefficients (r) between rainbow trout skin and muscle redness (a*-values) and oxidative stress response.

Oxidative Stress Response	Redness a*-Values	
	Muscle	Skin
Hepatic AX	0.85 **	0.62 **
Hepatic GR activity	ns	0.40 *
Hepatic OSI	0.36 *	0.27
Hepatic TBARS	−0.36 *	−0.34 *
Muscle TBARS	−0.42 *	−0.30 **
Hepatic gr mRNA level	0.45 **	0.39 *
Hepatic gclc mRNA level	0.36 *	0.25
Hepatic tr mRNA level	0.42 *	0.34 *
Hepatic g6pd mRNA level	0.38 *	0.35 *
Hepatic gckb mRNA level	0.36 *	0.24

* *p* < 0.05; ** *p* < 0.01.

## References

[1] Halliwell B., Gutteridge J.M.C. (2007). Free Radicals in Biology and Medicine.

[2] Lushchak V.I. (2011). Environmentally induced oxidative stress in aquatic animals. Aquatic. Toxicol..

[3] Rodriguez-Concepcion M., Avalos J., Bonet M.L., Boronat A., Gomez-Gomez L., Hornero-Mendez D., Limon M.C., Meléndez-Martínez A.J., Olmedilla-Alonso B., Palou A. (2018). A global perspective on carotenoids: Metabolism, biotechnology, and benefits for nutrition and health. Prog. Lipid. Res..

[4] Tacon A.G.J. (1981). Speculative review of possible carotenoid function in fish. Prog. Fish Cult..

[5] McGraw K.J., Hill G.E., McGraw K.J. (2006). Mechanics of Carotenoid-Based Coloration. Bird Coloration I: Mechanisms and Measurements.

[6] Choubert G., Storebakken T. (1989). Dose response to astaxanthin and canthaxanthin pigmentation of rainbow trout fed various dietary carotenoid concentrations. Aquaculture.

[7] Bjerkeng B., Peisker M., Von Schwartzenberg K., Ytrestøyl T., Åsgård T. (2007). Digestibility and muscle retention of astaxanthin in Atlantic salmon, *Salmo salar*, fed diets with the red yeast *Phaffia rhodozyma* in comparison with synthetic formulated astaxanthin. Aquaculture.

[8] Hama S., Uenishi A., Yamada T., Ohgita H., Tsuchiya E., Yamashita K. (2012). Scavenging of hydroxyl radicals in aqueous solution by astaxanthin encapsulated in liposomes. Biol. Pharm. Bull..

[9] Kurashige M., Okimasu E., Inoue M., Utsumi K. (1990). Inhibition of oxidative injury of biological membranes by astaxanthin. Physiol. Chem. Phys. Med. NMR.

[10] Amar E.C., Kiron V., Satoh S., Watanabe T. (2001). Influence of various dietary synthetic carotenoids on bio-defence mechanisms in rainbow trout, *Oncorhynchus mykiss* (Walbaum). Aquac. Res..

[11] Rahman M.M., Khosravi S., Chang K.H., Lee S. (2016). Effects of dietary inclusion of astaxanthin on growth, muscle pigmentation and antioxidant capacity of juvenile rainbow trout (*Oncorhynchus mykiss*). Prev. Nutr. Food Sci..

[12] Elia A.C., Prearo M., Dörr A.J.M., Pacini N., Magara G., Brizio P., Abete M.C. (2019). Effects of astaxanthin and canthaxanthin on oxidative stress biomarkers in rainbow trout. J. Toxicol. Environ. Health.

[13] Sallam A.E., Mansour A.T., Srour T.M., Goda A.M.A. (2017). Effects of different carotenoid supplementation sources with or without sodium taurocholate on growth, feed utilization, carotenoid content and antioxidant status in fry of the European seabass, *Dicentrarchus labrax*. Aquac. Res..

[14] Pham M.A., Byun H.G., Kim K.D., Lee S.M. (2014). Effects of dietary carotenoid source and level on growth, skin pigmentation, antioxidant activity and chemical composition of juvenile olive flounder *Paralichthys olivaceus*. Aquaculture.

[15] Wang Y.J., Chein Y.H., Pan C.H. (2006). Effect of dietary supplementation of carotenoid on survival, growth, pigmentation, and antioxidant capacity of characins, *Hyphessobrycon callistus*. Aquaculture.

[16] Pan C.H., Chien Y.H., Wang Y.J. (2011). Antioxidant defence to ammonia stress of characins (*Hyphessobrycon eques* Steindachner) fed diets supplemented with carotenoids. Aquac. Nutr..

[17] Rama S., Manjabhat S.N. (2014). Protective effect of shrimp carotenoids against ammonia stress in common carp, *Cyprinus carpio*. Ecotoxicol. Environ. Saf..

[18] Parolini M., Iacobuzio R., Possenti C.D., Bassano B., Pennati R., Saino S. (2018). Carotenoid-based skin coloration signals antioxidant defenses in the brown trout (*Salmo trutta*). Hydrobiologia.

[19] Ritola O., Tossavainen K., Kiuru T., Lindström-Seppä P., Mölsä H. (2002). Effects of continuous and episodic hyperoxia on stress and hepatic glutathione levels in one summer old rainbow trout (*Oncorhynchus mykiss*). J. Appl. Ichthyol..

[20] Wood C.M. (1991). Branchial ion and acid—base transfer in freshwater teleost fish: Environmental hyperoxia as a probe. Physiol. Zool..

[21] Pérez-Rodríguez L., Mougeot F., Alonso-Álvarez C. (2010). Carotenoid-based coloration predicts resistance to oxidative damage during an immune challenge. J. Exp. Biol..

[22] EFSA Panel on Additives and Products or Substances used in Animal Feed (2014). Scientific Opinion on the safety and efficacy of astaxanthin (CAROPHYLL^®^ Pink 10% CWS) for salmonids and ornamental fish. EFSA J..

[23] Folch J., Lees M.S., Stanley G.H.S. (1957). A simple method for the isolation and purification of total lipids from animal tissue. J. Biol. Chem..

[24] Good C.A., Kramer H., Somogyi M. (1933). The determination of glycogen. J. Biol. Chem..

[25] International Commission on Illumination (1976). Official Recommendations on Uniform Colour Space, Colour Difference Equations and Metric Colour Terms.

[26] García-de Blas E., Mateo R., Alonso-Alvarez C. (2015). Accumulation of dietary carotenoids, retinoids and tocopherol in the internal tissues of a bird: A hypothesis for the cost of producing colored ornaments. Oecologia.

[27] Koski P., Pakarinen M., Nakari T., Soivio A., Hartikainen K. (1999). Treatment with thiamine hydrochloride and astaxanthin for the prevention of yolk-sac mortality in Baltic salmon fry (M74 syndrome). Dis. Aquat. Org..

[28] Burk R.F., Trumble M.J., Lawrence R.A. (1980). Rat hepatic cytosolic GSH-dependent enzyme protection against lipid peroxidation in the NADPH microsomal lipid peroxidation system. Biochim. Biophys. Acta.

[29] Fontagné-Dicharry S., Lataillade E., Surget A. (2014). Antioxidant defense system is altered by dietary oxidized lipid in first-feeding rainbow trout (*Oncorhynchus mykiss*). Aquaculture.

[30] Lowry O.H., Rosebrough N.J., Farr A.L. (1951). Protein measurement with the Folin-phenol reagent. J. Biol. Chem..

[31] Fontagné-Dicharry S., Larroquet L., Dias K., Cluzeaud M., Heraud C., Corlay D. (2018). Effects of dietary oxidized fish oil supplementation on oxidative stress and antioxidant defense system in juvenile rainbow trout (*Oncorhynchus mykiss*). Fish Shellfish Immun..

[32] Pfaffl M.W. (2001). A new mathematical model for relative quantification in real-time RT-PCR. Nucleic Acids Res..

[33] Surai P.F., Fisinin V., Karadas F. (2016). Antioxidant systems in chick embryo development. Part 1. Vitamin E, carotenoids and selenium. Anim. Nutr..

[34] Amar E.C., Kiron V., Akutsu T., Satoh S., Watanabe T. (2012). Resistance of rainbow trout *Oncorhynchus mykiss* to infectious hematopoietic necrosis virus (IHNV) experimental infection following ingestion of natural and synthetic carotenoids. Aquaculture.

[35] Baker R.T.M., Pfeiffer A.M., Schöner F.J., Smith-Lemmon L. (2002). Pigmentation efficacy of astaxanthin and canthaxanthin in fresh-water reared Atlantic salmon, *Salmo salar*. Anim. Feed Sci. Technol..

[36] Kalinowski C.T., Robaina L., Fernandez-Palacios H., Schuchardt D., Izquierdo M.S. (2005). Effect of different carotenoid sources and their dietary levels on red porgy (*Pagrus pagrus*) growth and skin colour. Aquaculture.

[37] Pickering A.D., Pottinger T.G. (1989). Stress responses and disease resistance in salmonid fish: Effects of chronic elevation of plasma cortisol. Fish Physiol. Biochem..

[38] Barton B.A., Iwama G.K. (1991). Physiological changes in fish from stress in aquaculture with emphasis on the response and effects of corticosteroids. Ann. Rev. Fish Dis..

[39] Barton B.A. (2002). Stress in fishes: A diversity of responses with particular reference to changes in circulating corticosteroids. Integ. Comp. Biol..

[40] Van der Boon J., van den Thillart G.E., Addink A.D. (1991). The effects of cortisol on intermediary metabolism in teleost fish. Comp. Biochem. Physiol. Part A Physiol..

[41] Morales A.E., Cardenete G., Abellán E., García-Rejón L. (2005). Stress-related physiological responses to handling in the common dentex (*Dentex dentex* Linnaeus, 1758). Aquac. Res..

[42] Shoemaker C.A., Klesius P.H., Lim C., Yildirim M. (2003). Feed deprivation of channel catfish, *Ictalurus punctatus* (Rafinesque), influences organosomatic indices, chemical composition and susceptibility to Flavobacterium columnare. J. Fish Dis..

[43] Lim K.C., Yusoff F.M., Shariff M., Kamarudin M.S., Nagao N. (2019). Dietary supplementation of astaxanthin enhances hemato-biochemistry and innate immunity of Asian seabass, *Lates calcarifer* (Bloch, 1790). Aquaculture.

[44] Ursoniu S., Sahebkar A., Serban M.C., Banach M. (2015). Lipid profile and glucose changes after supplementation with astaxanthin: A systematic review and meta-analysis of randomized controlled trials. Arch. Med. Sci..

[45] Ikeuchi M., Koyama T., Takahashi J., Yazawa K. (2006). Effects of astaxanthin supplementation on exercise-induced fatigue in mice. Biol. Pharm. Bull..

[46] Aoi W., Naito Y., Takanami Y., Ishii T., Kawai Y., Akagiri S., Kato Y., Osawa T., Yoshikawa T. (2008). Astaxanthin improves muscle lipid metabolism in exercise via inhibitory effect of oxidative CPT I modification. Biochem. Biophys. Res. Commun..

[47] Page G.I., Russell P.M., Davies S.J. (2005). Dietary carotenoid pigment supplementation influences hepatic lipid and mucopolysaccharide levels in rainbow trout (*Oncorhynchus mykiss*). Comp. Biochem. Physiol. B.

[48] Storey K.B. (1996). Oxidative stress: Animal adaptations in nature. Braz. J. Med. Biol. Res..

[49] Berntssen M.H., Lundebye A.K., Hamre K. (2000). Tissue lipid peroxidative responses in Atlantic salmon (*Salmo salar* L.) parr fed high levels of dietary copper and cadmium. Fish Physiol. Biochem..

[50] Lushchak V.I., Bagnyukova T.V., Lushchak O.V., Storey J.M., Storey K.B. (2005). Hypoxia and recovery perturb free radical processes and antioxidant potential in common carp (*Cyprinus carpio*) tissues. Int. J. Biochem. Cell Biol..

[51] Lygren B., Hamre K., Waagbø R. (2000). Effect of induced hyperoxia on the antioxidant status of Atlantic salmon *Salmo salar* L. fed three different levels of dietary vitamin E. Aquac. Res..

[52] Brambilla F., Forchino A., Antonini M., Rimoldi S., Terova G., Saroglia M. (2009). Effect of dietary Astaxanthin sources supplementation on muscle pigmentation and lipid peroxidation in rainbow trout (*Oncorhynchus mykiss*). Ital. J. Anim. Sci..

[53] Liu F., Shi H.Z., Guo Q.S., Yu Y.B., Wang A.M., Lv F., Shen W.B. (2016). Effects of astaxanthin and emodin on the growth, stress resistance and disease resistance of yellow catfish (*Pelteobagrus fulvidraco*). Fish Shellfish Immunol..

[54] Nordgarden U., Ørnsrud R., Hansen T., Hemre G.I. (2003). Seasonal changes in selected muscle quality parameters in Atlantic salmon (*Salmo salar* L.) reared under natural and continuous light. Aquac. Nutr..

[55] Page G.I., Davies S.J. (2006). Tissue astaxanthin and canthaxanthin distribution in rainbow trout (*Oncorhynchus mykiss*) and Atlantic salmon (*Salmo salar*). Comp. Biochem. Physiol. Part A Mol. Integr. Physiol..

[56] Ritola O., Livingstone D.R., Peters L.D. (2002). Lindström-Seppä, P. Antioxidant processes are affected in juvenile rainbow trout (*Oncorhynchus mykiss*) exposed to ozone and oxygen supersaturated water. Aquaculture.

[57] Olsvik P.A., Lie K.K., Jordal A.E.O., Nilsen T.O., Hordvik I. (2005). Evaluation of potential reference genes in real-time RT-PCR studies of Atlantic salmon. BMC Mol. Biol..

[58] Olsvik P.A., Kristensen T., Waagbø R., Rosseland B.O., Tollefsen K.E., Toften H. (2006). Effects of hypo- and hyperoxia on transcription levels five stress genes and the glutathione system in liver of Atlantic cod *Gadus morhua*. J. Exp. Biol..

[59] Salas-Leiton E., Cánovas-Conesa B., Zerolo R., López-Barea J., Cañavate J.P., Alhama J. (2009). Proteomics of Juvenile Senegal Sole (*Solea senegalensis*) Affected by Gas Bubble Disease in Hyperoxygenated Ponds. Mar. Biotechnol..

[60] Hoffmann C., Dietrich M., Herrmann A.K., Schacht T., Albrecht P., Methner A. (2017). Dimethyl fumarate induces glutathione recycling by upregulation of glutathione reductase. Oxid. Med. Cell Longev..

[61] Cappellini M.D., Fiorelli G. (2008). Glucose-6-phosphate dehydrogenase deficiency. Lancet.

[62] Nóbrega-Pereira S., Fernandez-Marcos P.J., Brioche T., Gomez-Cabrera M.C., Salvador-Pascual A., Flores J.M., Serrano M. (2016). G6PD protects from oxidative damage and improves healthspan in mice. Nat. Commun..

[63] Tang H.Y., Ho H.Y., Wu P.R., Chen S.H., Kuypers F.A., Cheng M.L. (2015). Inability to maintain GSH pool in G6PD-deficient red cells causes futile AMPK activation and irreversible metabolic disturbance. Antioxid. Redox Signal..

[64] Wu G., Fang Y.Z., Yang S., Lupton J.R., Turner N.D. (2004). Glutathione metabolism and its implications for health. J. Nutr..

[65] Steullet P., Cabungcal J.H., Kulak A., Kraftsik R., Chen Y., Dalton T.P. (2010). Redox dysregulation affects the ventral but not dorsal hippocampus: Impairment of parvalbumin neurons, gamma oscillations, and related behaviors. J. Neurosci..

[66] Dose J., Matsugo S., Yokokawa H., Koshida Y., Okazaki S., Seidel U., Eggersdorfer M., Rimbach G., Esatbeyoglu T. (2016). Free radical scavenging and cellular antioxidant properties of astaxanthin. Int. J. Mol. Sci..

[67] Nordberg J., Arnér E.S. (2001). Reactive oxygen species, antioxidants, and the mammalian thioredoxin system. Free Radic. Biol. Med..

